# Supplementary material for: Content-rich biological network constructed by mining PubMed abstracts
Source: BMC Bioinformatics. 2004 Oct 8;5:147. doi: 10.1186/1471-2105-5-147 (PMC528731; doi:10.1186/1471-2105-5-147)
Supplement: Additional File 2 — The original results of the above study (non-essential files are deleted to keep the file size under the limit set by BMC bioinformatics). [file 1471-2105-5-147-S2.bz2 › chilibotAdditionalFile2/dip10/198ID8652557E683/html/ERCC2_GTF2H1.html]

 


 **ERCC2** and **GTF2H1** 
  
Found 85 abstracts in PubMed, retrieved 10.  
 

 What does Google say? 
 PDF only 
| .edu only 

---

**Interactive relationship** (e.g. stimulation, inhibition, etc)

**Inhibitory relationship**- Moreover, when XPD  [ **ERCC2** ]  mutations prevent interaction with the p44 subunit of TFIIH  [ **GTF2H1** ] , transactivation directed by certain nuclear receptors is inhibited, regardless of TTD versus XP phenotype, thus explaining the overlapping symptoms.  Ref: 12820975 Mol Cell, 2003
**Neutral relationship**- Trichothiodystrophy TTD is a rare hereditary multisystem disorder associated with defects in nucleotide excision repair NER as a consequence of mutations in XPD  [ **ERCC2** ] , XPB or TTDA, three genes that are all related to TFIIH  [ **GTF2H1** ] , the multiprotein complex involved in NER and transcription.  Ref: 12393803 Hum Mol Genet, 2002
- We also show that mutations in CSB, as well as in XPB and XPD  [ **ERCC2** ]  genes, all of which confer CS, disturb the RNA pol I TFIIH  [ **GTF2H1** ]  interaction within the CSB IP 150.  Ref: 12419226 Mol Cell, 2002
- General transcription factor IIH TFIIH  [ **GTF2H1** ]  consists of nine subunits cyclin dependent kinase 7 Cdk7, cyclin H and MAT1 forming the Cdk activating kinase or CAK complex, the two helicases Xpb Hay and Xpd  [ **ERCC2** ] , and p34, p44, p52 and p62 refs 1 3.  Ref: 12853965 Nature, 2003

**Non-interactive relationship** (e.g. studied together, co-existance, homology, etc.)

- Mutations in XPB and XPD  [ **ERCC2** ]  TFIIH  [ **GTF2H1** ]  helicases have been related with three hereditary human disorders xeroderma pigmentosum, Cockayne syndrome, and trichothiodystrophy.  Ref: 12221129 Mol Biol Cell, 2002
- Regulation of cisplatin resistance and homologous recombinational repair by the TFIIH  [ **GTF2H1** ]  subunit XPD  [ **ERCC2** ] .  Ref: 12359753 Cancer Res, 2002
- We conclude that the severity of the clinical features in TTD patients and the clinical outcome of differentially mutated XPD  [ **ERCC2** ]  proteins is likely to depend both on the effects that each mutation has on the stability of TFIIH  [ **GTF2H1** ]  and on the transcriptional activity of the residual TFIIH  [ **GTF2H1** ]  complexes.  Ref: 12393803 Hum Mol Genet, 2002
- We have also measured TFIIH  [ **GTF2H1** ]  levels in cells in which different mutations in the XPD  [ **ERCC2** ]  gene are associated with clinical symptoms not of TTD but of the highly cancer prone disorder xeroderma pigmentosum XP.  Ref: 12393803 Hum Mol Genet, 2002
- Here we show that the Drosophila TFIIH  [ **GTF2H1** ]  component Xpd  [ **ERCC2** ]  negatively regulates the cell cycle function of Cdk7, the CAK activity.  Ref: 12853965 Nature, 2003
- We have recently completed screening of the National Cancer Institute human tumor cell line panel and demonstrated that among four nucleotide excision repair proteins XPA, XPB, XPD  [ **ERCC2** ] , and ERCC1, only the TFIIH  [ **GTF2H1** ]  subunit XPD  [ **ERCC2** ]  endogenous protein levels correlate with alkylating agent drug resistance.  Ref: 12359753 Cancer Res, 2002
- In this report, we demonstrate an association of a C terminal domain of human RAD52 amino acids 302 418 with the XPB and XPD  [ **ERCC2** ]  subunits of transcription factor TFIIH  [ **GTF2H1** ]  and RNA polymerase II RNAPII.  Ref: 12372413 Biochem Biophys Res Commun, 2002
- Three polypeptides, primarily positioned 5 to the damage, are in close physical proximity to the psoralen lesion and thus are cross linked to the damaged DNA these proteins are RPA70, RPA32, and the XPD  [ **ERCC2** ]  subunit of TFIIH  [ **GTF2H1** ] .  Ref: 12138203 Mol Cell Biol, 2002
- The antibody study indicated that they thus altered the conformation of one side of TFIIH  [ **GTF2H1** ] , consisting of p44, XPD  [ **ERCC2** ] , and Cdk activating kinase subunits, that is essential for the transition stage.  Ref: 12665589 Mol Cell Biol, 2003
